# Safety and Efficacy of Low-Level Laser Therapy in Autoimmune Thyroiditis: Long-Term Follow-Up Study

**DOI:** 10.1155/2018/8387530

**Published:** 2018-11-04

**Authors:** Danilo Bianchini Höfling, Maria Cristina Chavantes, Carlos Alberto Buchpiguel, Giovanni Guido Cerri, Suemi Marui, Paulo Campos Carneiro, Maria Cristina Chammas

**Affiliations:** ^1^Ultrasound Unit, Department of Radiology, University of Sao Paulo Medical School, Hospital das Clínicas, 05403-000 São Paulo, SP, Brazil; ^2^Universidade Nove de Julho, Post-Graduation, 01504-001 São Paulo, SP, Brazil; ^3^Radiology Institute (InRad); Department of Radiology, University of Sao Paulo Medical School, Hospital das Clínicas, 05403-000 São Paulo, SP, Brazil; ^4^Thyroid Unit, Department of Endocrinology and Metabolism, University of Sao Paulo Medical School, Hospital das Clínicas, 05403-000 São Paulo, Brazil; ^5^Department of Pathology, University of Sao Paulo Medical School, Hospital das Clínicas, 05403-000 São Paulo, Brazil

## Abstract

**Introduction:**

A randomized clinical trial (RCT) was performed to evaluate the efficacy of low-level laser therapy (LLLT) for hypothyroidism induced by chronic autoimmune thyroiditis (CAT).

**Objective:**

The objective was to assess the safety and actions of LLLT 6 years after completion of the RCT.

**Materials and Methods:**

Forty-three participants were invited to participate in this study 6 years after completion of the RCT. Twenty-five were subjected to LLLT (group L), and 18 were subjected to placebo (group P). Primary outcome measure: frequency of thyroid nodules, which were subjected to fine-needle aspiration biopsy. Secondary outcome measures: dose of levothyroxine required to treat hypothyroidism, thyroid peroxidase antibodies (anti-TPO), and anti-thyroglobulin antibodies (anti-Tg).

**Results:**

In group L, a nodule was observed in three patients, who all had a Bethesda II classification. In group P, a nodule was also observed in three patients, with two classified as Bethesda II and one as Bethesda III. The levothyroxine dose required by group L was significantly lower than that required by group P (*P* = 0.002). The anti-TPO and anti-Tg levels did not differ between the groups.

**Conclusion:**

LLLT, by the methods described, has been shown to be safe for the treatment of hypothyroidism resulting from CAT. This trial is registered with ClinicalTrials.gov Identifier: NCT02240563.

## 1. Introduction

Low-level laser therapy (LLLT), or photobiomodulation (PBM), is a simple, noninvasive procedure without ionizing radiation in which red or infrared light is used. Its action has already been studied in several tissues, including thyroid tissues. Electron microscopy studies have shown that the use of LLLT does not cause damage to the thyroid parenchyma of mice [1–3]. Furthermore, in rats, LLLT ameliorated the damaging effect of gamma irradiation on the gland [4].

In humans, we initially assessed LLLT in patients with hypothyroidism caused by chronic autoimmune thyroiditis (CAT) in a pilot study [5]. We then studied patients under the same conditions using a randomized clinical trial (RCT) [6–8]; the results showed a reduction in the levothyroxine (LT4) doses required to treat hypothyroidism, and 47.8% of the patients did not need to take LT4 during the 9 months of follow-up, suggesting an improvement in gland function. LLLT has a regenerative effect on various tissue types [9]. Therefore, LLLT could also act in the regeneration of thyroid follicular cells and explain the improvement of thyroid function verified in the RCT. In addition, we observed an increase in echogenicity by real-time computerized grayscale histogram analysis in the patients subjected to LLLT. The thyroid follicular structure, which represents the main acoustic interface of the gland, is able to provide ideal conditions for reflection of the intense ultrasound echoes to the equipment probe. In the case of CAT, the follicle destruction and the presence of lymphocytic infiltration promote scattering of these waves, which reduces sound reflection and results in hypoechogenicity [10, 11]. Hence, the augmentation of echogenicity observed in the RCT suggests partial regeneration of follicular structure and/or a decrease in lymphocytic infiltration.

The reduction of thyroid peroxidase antibodies (anti-TPO) was also noted in such patients [6], indicating a decrease in the autoimmune process against the gland.

Although the initial results appear promising, the safety and long-term actions of LLLT on thyroid tissue in CAT patients are unknown. CAT is one of the causes of thyroid nodule formation [12]. In addition, reports have suggested that CAT may be associated with a significantly increased frequency of well-differentiated thyroid carcinoma [13]. Thus, it is particularly important to evaluate the influence of LLLT on the frequency of the development of thyroid nodules.

Since the actions of LLLT on thyroid function and antithyroid antibodies are likely transient, subsequent applications will be required depending on several individual factors, such as the intensity of the autoimmune response and the degree of the parenchymal lesion.

With such considerations, the objective of this research was to assess the safety and effects of LLLT 6 years after completion of RCT [6] by investigating thyroid nodules, the LT4 dose required to treat hypothyroidism, the concentrations of anti-TPO and anti-thyroglobulin antibodies (anti-Tg), and the color Doppler ultrasound (CDU) images.

## 2. Materials and Methods

This is the long-term follow-up of 43 patients with CAT-induced hypothyroidism included in our RCT performed between March 2006 and March 2009 [6]. Participants were under treatment with adequate and stable doses of LT4, had high anti-TPO and/or anti-Tg concentrations, and had a thyroid parenchyma with reduced echogenicity and a diffusely heterogeneous texture without nodules in all CDU examinations. These patients were randomized to receive 10 sessions of LLLT (L group) or placebo (P group), twice a week, for a total of 5 weeks of treatment [6]. The L group was treated with a continuous-wave diode laser device (infrared laser of 830 nm) at an output power of 50 mW, a fluence of 707 J/cm^2^ (40 s at each point of application), and an irradiance of 17.68 W/cm^2^. The P group was subjected to the same method and equipment using an ordinary red light with an output power of 0.1 mW, a fluence of 1.41 J/cm^2^, and an irradiance of 0.03536 W/cm^2^ [6].

Twenty-three patients were subjected to LLLT, and 20 to placebo LT4 were discontinued 30 days after the end of the interventions. From that moment, total T3, total T4, free T4 (fT4), and thyrotropin (TSH) measurements were performed at 30, 60, 90, 180, and 270 days to evaluate the presence of hypothyroidism and reintroduce appropriate doses of LT4, according to preestablished criteria [6].

Two of the placebo-treated patients during the RCT were then subjected to LLLT. Thus, in total, 25 patients were treated with LLLT and 18 with placebo.

**Figure 1:**
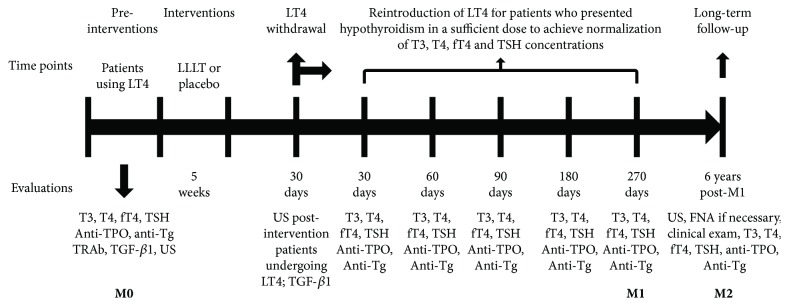
Study design.

As of January 2014, we began calling patients to adjust the LT4 supplementation doses. In September 2014, patients in the L and the P groups began to be reassessed by the time they reached 6 years after completing the RCT (Figure 1). To compare the results of the RCT and those of this study, three different moments were considered: before the interventions performed in the RCT (M0); at the follow-up to determine the outcome of each RCT participant, that is, 270 days after suspension of LT4 (M1); and during the present study (M2) performed 6 years after M1. At M2, clinical evaluations, biochemical measurements, and CDU were performed for each patient (Figure 1).

This study was conducted at the Radiology Institute of the Clinics Hospital, University of Sao Paulo Medical School (Hospital das Clínicas-Faculdade de Medicina da Universidade de São Paulo (HC-FMUSP)). HC-FMUSP's Research Ethics Committee approved this study and the patient consent forms. All the patients signed the consent forms voluntarily.

### 2.1. Inclusion Criteria

The inclusion criteria are patients who participated in the RCT (NCT01129492).

### 2.2. Exclusion Criteria

The exclusion criteria include (a) RCT patients subjected to radioiodine therapy of the thyroid or surgical intervention of the gland and/or neck during the period of follow-up; (b) RCT patients using immunosuppressants, immunostimulants, or drugs that interfere with the production, transport, and metabolism of thyroid hormones (e.g., corticosteroids, lithium, and amiodarone); and (c) RCT patients who were pregnant.

### 2.3. Study Design

Six years after M1, clinical evaluation, biochemical measurements, and CDU were performed for each patient (Figure 1).

Prior to the assessment of the patients, appropriate and stable LT4 replacement doses for each patient were adjusted by a physician from the Thyroid Outpatient Clinic of the HC-FMUSP who was blinded to the treatment assignments. The adjustments to LT4 supplementation doses were determined according to the same criteria utilized for the RCT patients: increase of 25 *μ*g/day in LT4 when the TSH was >4.5 *μ*U/mL and <10 *μ*U/mL; increase of 50 *μ*g/day in LT4 when the TSH was ≥10 *μ*U/mL; and reduction of 25 *μ*g/day in LT4 when the TSH was <0.4 *μ*U/mL.

Then, as patients reached 6 years after their participation in the RCT, they were subjected to blood collection for biochemical measurements and to CDU.

The CDU parameters were used both to identify the presence of thyroid nodules and to investigate the autoimmune inflammatory process. Before the LLLT and placebo interventions, none of the patients had evidence of nodules by CDU.

For the assessment of LLLT safety in the induction of thyroid cancer, fine-needle aspiration (FNA) biopsy was planned for all nodules identified by CDU. The test was interpreted by a cytopathologist experienced in thyroid pathology who was blinded to the treatment groups.

Anti-TPO and anti-Tg levels were measured to estimate the autoimmune response. The dose of LT4 required to maintain normal (or near normal) serum concentrations of total T3, total T4, fT4, and TSH was also determined for patients in both groups.

### 2.4. Outcome Measures

The primary outcome measure was LLLT safety estimated by the frequency of benign and malign nodules detected at M2. The secondary outcome measures were the LT4 dose required to achieve normal total T3, total T4, fT4, and TSH concentrations, the serum concentrations of anti-TPO and anti-Tg, and the CDU parameters at M2.

### 2.5. Color Doppler Ultrasound Study

CDU was performed by an investigator who was blinded to the treatment groups. The exam was performed with the patient in the supine position and a cushion under their shoulders with their neck hyperextended.

### 2.6. Equipment

A high-resolution IU22™ device (Philips Medical Systems®, Bothell, WA, USA) attached to a broadband linear probe (5–12 MHz) was employed for the B-mode and color flow Doppler thyroid parameters analyses.

### 2.7. Gray Scale (B-Mode) Sonography

The size, volume, shape, echogenicity, and echotexture of the gland were examined as well as the presence or absence of thyroid nodules. The echogenicity was subjectively analyzed by comparing the intensity of the echoes from the thyroid with those from the sternocleidomastoid and prethyroid muscles. The thyroid parenchyma echo intensity was divided into three categories: normal, increased, or reduced echogenicity. The echotexture was categorized as either diffusely homogeneous or heterogeneous. The volume (cubic centimeters) of each lobe and isthmus was calculated by the formula: length (centimeters) × width (centimeters) × depth (centimeters) × 0.528. We adopted reference values of 6–16 cm^3^ [14].

When nodules were identified, their characteristics, such as dimensions, echogenicity, shape, border regularity, solid/mixed, presence of halo, presence of calcifications and microcalcifications, vascularization, and resistivity indices of the intranodular arteries, were examined.

The CDU images were obtained using color and pulsed Doppler imaging.

### 2.8. Color Doppler

The transducer was lightly supported on the skin, without pressing of the skin to avoid an underestimation of the vascularization due to the compression of the blood vessels.

The device was configured as follows: color Doppler gain of approximately 80%; wall filter (WF) low and pulse-repetition frequency (PRF) of approximately 750 Hz; and velocity scale of 5.0 cm/s. The isthmus vascularization was not assessed. The color gain was adjusted up to the highest possible level that was not associated with image saturation artifact. All of the two-dimensional images were recorded at the time of the greatest visible flow, corresponding to the peak systolic velocity (PSV) of blood flow.

The thyroid vascularization pattern (TVP) was classified into four categories, as previously planned [6]: pattern 0—the vascularization was decreased and was limited to the main peripheral arteries, which had reduced signals; pattern I—the vascularization was limited to the main peripheral thyroid arteries, which exhibited the usual signals, whereas only signals of vascularization focal points with either a scattered distribution or a localized presence were found in the interiors of the nodules; pattern II—the vascularity clearly increased and had a scattered distribution; and pattern III—the vascularization was markedly increased and had a diffuse and homogeneous distribution, including the so-called “thyroid inferno” pattern.

We assigned vascularization patterns from 0 to III to the right and left lobes of each patient's thyroid, that is, a total of 86 lobes.

### 2.9. Pulsed Doppler

The parameters used for the pulsed Doppler were the same as those used for the color Doppler. For PSV and resistive index (RI) measurements, both the right and left inferior thyroid arteries (ITAs) were identified, and the sampling volume was adjusted to 1 mm at the center of these vessels. The Doppler angle was always corrected to values smaller than 60°. Both the right and the left ITAs were examined in the oblique axial plane. The values of the peak systolic velocity of the inferior thyroid arteries (ITA-PSV) and the resistive index of the inferior thyroid arteries (ITA-RI) were obtained from pulsed Doppler analysis. The mean values of the right and left ITA-PSV and ITA-RI were used as representative variables for the statistical analysis.

### 2.10. Thyroid Nodule Cytopathology

Ultrasound-guided FNA biopsies were performed in all identified thyroid nodules. The IU22™ device (ATL/Philips®; Bothell, Washington, USA) with a 7–12 MHz linear probe was used to guide the procedures. The aspiration procedure was performed by means of the “mixed sampling technique” in which the operator used his or her wrist to move the needle up and down for a few seconds [15]. A 23-gauge needle attached to 10 mL plastic syringe coupled to a device to induce negative pressure was used to carry out the procedure. Three smears were obtained from each nodule and were sent for cytopathological diagnosis. All cytological smears were evaluated by one expert cytopathologist according to the Bethesda System for Reporting Thyroid Cytology [16].

### 2.11. Biochemical Measurements

Venous blood samples were obtained in the morning, after an overnight fast and before ingesting any medication on the same day that the CDU was performed.

The serum concentrations of total T3, total T4, fT4, TSH, anti-TPO, and anti-Tg were measured through the chemiluminescence method, using total T3, total T4, fT4, TSH, anti-TPO, and anti-Tg ADVIA Centaur® XP Immunoassay System kits (Siemens Healthcare Diagnostics Inc., Tarrytown, NY, USA). All biochemical exams were performed by the High Diagnostic Quality Laboratory (Laboratório Alta Excelência Diagnóstica-DASA Group, São Paulo, SP, Brazil).

The reference values, analytical sensitivities, intra-assay coefficients of variations, and inter-assay coefficients of variations for the aforementioned assays were the following, respectively: (A) total T3 = 70–220 ng/mL, 20 ng/dL, 2.22%, and 1.15%; (B) total T4 = 5.1–13.5 *μ*g/dL, 0.3 *μ*g/dL, 2.04%, and 2.98%; (C) fT4 = 0.7–1.8 ng/dL, 0.1 ng/dL, 2.7%, and 2.94%; (D) TSH = 0.4–4.3 *μ*IU/mL (15–61 years of age) or 0.4–5.8 *μ*IU/mL (over 61 years of age), 0.008 *μ*IU/mL, 2.02%, and 1.55%; (E) anti-TPO < 60 IU/mL, 1.0 IU/mL, 4.1%, and 3.1%; and (F) anti-Tg < 60 IU/mL, 1.0 IU/mL, 4.3%, and 3.0%. When the serum concentrations of anti-TPO and anti-Tg were below or above the detection limit, the corresponding minimum and maximum values were adopted as being representatives for the statistical analysis.

### 2.12. Statistical Analysis

Statistical analysis was conducted using SPSS® software (version 17.0-SPSS Inc., IBM® Headquarters Company, Chicago, IL, USA).

The baseline clinical data and the outcomes are presented as the mean ± standard deviation (SD). For the variables with a parametric distribution, the *t*-test was used for independent samples (not paired), and/or the *t*-test for paired samples was used. For nonparametric variables, the Mann–Whitney *U* test was used for independent samples (unpaired), and/or the Wilcoxon test was used for paired samples. The chi-squared and binomial tests were used for comparisons of proportions. Two-sided *P* values < 0.05 were considered statistically significant (^∗^).

## 3. Results

Between September 2014 and March 2016, the 43 RCT participants were evaluated in this study, and there was no loss of participants (Table 1). One of the patients who underwent LLLT refused to have his/her blood collected on the day his/her US was performed. Thus, his/her biochemical data were not included in the statistical analysis.

### 3.1. Primary Outcome Measure

The safety of LLLT was assessed by the frequency of thyroid nodules and their nature (benign or malignant). Of the 25 patients subjected to LLLT, three presented a nodule in the US. Of these three, each had a thyroid nodule. The three nodules were classified as category II of the Bethesda system. In the group of 18 placebo-treated patients, three showed nodules, each with a thyroid nodule (Table 2). Of the three nodules observed, two were classified as category II and one as category III of the Bethesda system.

### 3.2. Secondary Outcome Measures

The long-term effects of LLLT on thyroid function, antithyroid antibodies, and CDU parameters were assessed by comparing independent samples (group L versus group P at a given time) and/or paired samples (before and after intragroup). The two patients who were part of group P in the RCT and were then subjected to LLLT (i.e., they moved to group L) were excluded from the paired analysis.

The LT4 dose required to achieve normal total T3, total T4, fT4, and TSH concentrations, the anti-TPO and anti-Tg levels, and the US parameters were assessed at M2.

#### 3.2.1. Dose of LT4

The mean LT4 dose (*μ*g/day and *μ*g/kg/day) required to obtain normal (or near normal) mean concentrations of total T3, total T4, fT4, and TSH in the present study (M2) was significantly lower in group L than in group P (Table 3). There was no statistically significant difference in the concentrations of total T3, total T4, fT4, and TSH, the body mass (weight), and the BMI between both groups at M2 (Tables 1 and 3).

**Figure 2:**
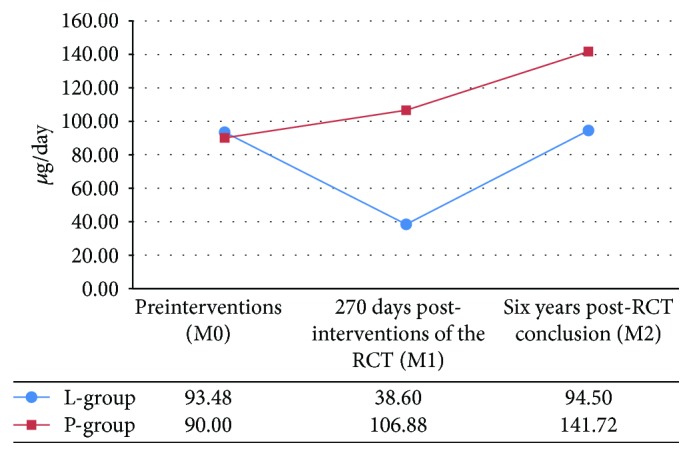
Replacement doses of LT4 (*μ*g/day) required by the patients of the L and P groups at M0, M1, and M2.

The paired analysis showed that there was no statistically significant difference between the mean dose of LT4 (*μ*g/day) used by the L group at M0 (93.48 ± 44.09 *μ*g/day) and M2 (91.28 ± 42.99 *μ*g/day; *P* = 0.696; Figure 2), whereas in group P, the dose used at M0 (88.89 ± 42.20 *μ*g/day) was significantly lower than that used at M2 (141.72 ± 52.62 *μ*g/day; ^∗^*P* < 0.001; Figure 2). In group L, the dose of LT4 at M2 (91.28 ± 42.99 *μ*g/day) was significantly higher than that at M1 (38.59 ± 46.75 *μ*g/day; ^∗^*P* < 0.001; Figure 2), whereas in group P, the dose of LT4 was also higher at M2 (141.72 ± 52.62 *μ*g/day) than at M1 (107.64 ± 50.94 *μ*g/day, ^∗^*P* = 0.001; Figure 2).

The mean dose of LT4 (*μ*g/kg/day) showed similar results in the paired analysis: group L at M0 (1.32 ± 0.60 *μ*g/kg/day) versus group L at M2 (1.23 ± 0.59 *μ*g/kg/day, *P* = 0.375); group P at M0 (1.18 ± 0.45 *μ*g/kg/day) versus group P at M2 (1.86 ± 0.53 *μ*g/kg/day, ^∗^*P* < 0.001). In group L, the dose of LT4 at M2 (1.23 ± 0.59 *μ*g/kg/day) was significantly higher than that at M1 (0.53 ± 0.64 *μ*g/kg/day; ^∗^*P* < 0.001), while in group P, the dose of LT4 was also significantly higher at M2 (1.86 ± 0.53 *μ*g/kg/day) than at M1 (1.41 ± 0.50 *μ*g/kg/day; ^∗^*P* < 0.001).

#### 3.2.2. Autoantibodies

At M2, the anti-TPO concentrations were not significantly different between group L (474.10 ± 475.35 IU/mL) and group P (702.67 ± 575.01 IU/mL; *P* = 0.166). The anti-Tg concentrations did not differ significantly between group L (176.80 ± 156.58 IU/mL) and group P (319.41 ± 298.90 IU/mL, *P* = 0.078). Paired analysis of both the anti-TPO and anti-Tg concentrations and positivity was not performed because the analysis kits used to measure the autoantibodies at M0 and M1 were different from those employed at M2.

#### 3.2.3. CDU Parameters

As at M0 and M1, all patients in both groups continued to exhibit thyroid parenchyma with reduced echogenicity and a diffusely heterogeneous texture at M2. The volume values did not differ significantly between the two groups at M2 (Table 3). However, at this moment, the proportion of patients with a normal volume was significantly higher in group L than in group P (Table 4).

The frequency of a normal TVP of the thyroid lobes in groups L and P did not differ significantly at M2 (Table 5). The right and left ITA-PSV and ITA-RI mean also did not differ significantly between the two groups at M2 (Table 3).

## 4. Discussion

Many studies have provided an impressive amount of information on the mechanisms of action of LLLT. Recently, the term LLLT has been replaced by the more appropriate term photobiomodulation (PBM) therapy.

LLLT (infrared) actions result from the interaction of light with endogenous photoacceptors during irradiation. The effects of infrared light come from the interaction with at least two main photoacceptors, including cytochrome c oxidase (unit IV in the mitochondrial respiratory chain) and intracellular water [9, 17]. One of the main hypotheses is that the photons dissociate inhibitory nitric oxide from the cytochrome c oxidase, which promotes increases in electron transport, mitochondrial membrane potential, and adenosine triphosphate (ATP) concentration [18]. Next, many signaling pathways are activated through Ca^2+^ release from intracellular stores, which increases the concentrations of cyclic adenosine monophosphate (cAMP), reactive oxygen species (ROS), nitric oxide (NO), and inositol phosphates, leading to the activation of transcription factors [9, 17, 18]. These factors can induce increased expression of genes related to the synthesis or release of many molecules, such as growth factors, interleukins, cytokines, anti-apoptotic proteins, and antioxidant enzymes. Such molecules are responsible for cell proliferation/differentiation, regeneration of several types of tissues, and modulation of immunological responses [9, 17, 18]. Our hypothesis is that such actions also occur during irradiation of thyroid parenchyma, thus promoting cell regeneration and modulation of autoimmune response.

The RCT showed evidence that LLLT promoted the following: (a) an improvement in thyroid function, as indicated by the reduction in the dose of LT4 used to treat hypothyroidism; (b) a reduction in serum anti-TPO concentrations; and (c) improvement in the echogenicity, volume, and thyroid vascularization pattern in CDU [6, 7]. Such results suggest that LLLT may be an interesting alternative for the treatment of CAT-induced hypothyroidism. However, the long-term effects and safety of this approach are unknown.

The infrared laser (830 nm) used in the RCT did not have ionizing radiation; therefore, it was not expected that there would be an increase in the frequency of nodules, including malignant ones, in the group that underwent LLLT.

As planned, the patients selected for the RCT did not present thyroid nodules. In the present study (6 years later), 6 thyroid nodules were observed in 43 patients, which corresponds to an incidence greater than double that observed in the general population [12]. This finding enforces the existence of an association between thyroid nodular disease and CAT [19].

In the current study, the proportion of nodules was similar between the L and the P groups. No malignant nodules were observed, and the only nodule of indeterminate nature (Bethesda III) was from a patient in group P. These results show that LLLT had no effect on the formation of benign and malignant thyroid nodules.

Although the use of LLLT (infrared) on thyroids with nodules has not yet been studied, ultrasound-guided interstitial photocoagulation with a high-power laser (infrared) has been evaluated by several authors for the treatment of benign thyroid nodules beginning in the 2000s [20–22]. Such studies provide indirect information on the safety of infrared LLLT for benign thyroid nodules. When a high-power laser beam interacts with the tissue, a higher concentration of energy occurs at the point of contact between the fiber and the tissue (photothermic effect). As the laser light moves away from this point, there is intense energy decay due to multiple scattering and absorption, which will reach the periphery of the treated area at very low levels compatible with those of the LLLT. This phenomenon is known as the *α*- or residual effect of the laser [23]. Although there is energy decay, the wavelength does not change. It may be noted that the same wavelength can promote both tissue destruction (photothermic effect) and regeneration (LLLT), depending on the power applied. Long-term studies have demonstrated that the use of high-power lasers is safe for the treatment of benign thyroid nodules [24–26]. Thus, we believe that when a high-power laser is used to promote photocoagulation (photothermic effect) within a thyroid nodule, LLLT actions occur at the periphery or surrounding the nodule due to energy decay. Since wavelengths that do not have ionizing radiation were employed in both cases, we suppose that the use of infrared LLLT in patients with benign nodules might be equally safe.

The dose of LT4 required to treat hypothyroidism may indirectly indicate the functional capacity of the thyroid follicular cells. In the RCT, the L group had a reduction in the LT4 dose, but there was an increase in the P group [6].

In the present study, the dose of LT4 used for the L group was lower than that used for the P group. In the paired analysis, the dose of LT4 used for the L group was similar to that used before LLLT and higher than that used at M1 of the RCT (Figure 2). At M1, the required dose of LT4 was significantly lower in the L group than in the P group (Figure 2); this difference was possibly associated with the tissue regeneration action promoted by LLLT already demonstrated in other tissues [9]. However, as we predicted, this supposed effect on follicular cells is limited in time, since the required dose of LT4 at M2 returned to the values used before LLLT. This result suggests that new LLLT applications would be needed over time. However, for this purpose, demonstrating safety is indispensable.

In group P, when comparing the preplacebo moments, 270 days postplacebo follow-up and the present study, a progressive increase in the required dose of LT4 was observed, suggesting gradual deterioration of follicular cell function as a result of the lesion promoted by an autoimmune process (Figure 2). In this case, it is postulated that the absence of the LLLT regenerative stimulus of the follicular cells gave rise to a gradual injury promoted by the autoimmune process, which led to an increase in LT4 needs. This is possibly the reason why in the present study, the dose of LT4 used by the P group remained higher than that used by the L group.

High anti-TPO and anti-Tg levels indicate the presence of autoimmunity against the thyroid [27]. At the end of the RCT, the anti-TPO concentrations were lower in the L group than in the P group [6]; however, in the present study, such concentrations showed no significant difference. These results indicate that the reduction in post-LLLT anti-TPO has a limited effect over time but could be extended for a longer period through additional interventions. In the present study, the anti-Tg levels did not differ between the L and the P groups, while in the RCT, there was only a tendency for anti-Tg levels to decrease in the L group [6], indicating that LLLT, as it was employed, had no significant influence on anti-Tg levels.

At M2, although the mean thyroid volume did not differ between the two groups, the proportion of patients with a normal volume was higher in the L group than in the P group. A similar result was also observed for the CDU performed 30 days RCT postintervention. It is possible that the effects of LLLT on the inflammatory process and tissue regeneration may have contributed to this outcome.

The US performed 30 days RCT postintervention showed that the proportion of patients with a normal TVP was higher in the L group than in the P group [7], but in the present study, this proportion did not differ significantly. This finding indicates that the effects of LLLT on the improvement of TVP are also transient. In the pulsed Doppler US, both the mean ITA-PSV and the mean ITA-RI did not differ between the two groups. The results obtained in the RCT for these parameters were similar.

Some limitations of this study may be mentioned. The vascularization was evaluated by means of a subjective method, with classification of the vascularization into four different patterns. Although the evaluation of TVP was performed by a single experienced examiner, there may be intraobserver variations in interpretation in the case of borderline patterns. However, two separate analyses by two independent investigators could also be employed to improve this evaluation. The use of different anti-TPO and anti-Tg measurement kits during the RCT and the present study made comparative analysis impossible at those moments. The safety analysis was based on a small sample of patients. Therefore, further research is needed to reinforce these results.

In summary, the results showed that LLLT (infrared laser) is safe when used for the treatment of patients with CAT without nodules in the parenchyma, as there was no increase in the frequency of nodular lesions. There was also no worsening of autoimmunity, as the concentrations of antithyroid antibodies were similar in both groups. Further, thyroid function, as estimated indirectly by the dose of LT4 required to treat hypothyroidism, was better in the patients treated with LLLT. As we anticipated, the effects of LLLT are transient, and thus, new therapy sessions will be necessary over time to maintain the obtained RCT results. The RCT results showed that the effects of LLLT persisted for at least 11 months.

The importance of safety results should be emphasized not only for LLLT maintenance sessions but also mainly to enable the future assessment of the efficacy of LLLT in patients in the early stages of the disease, such as those with subclinical hypothyroidism. In our opinion, this will be the ideal time for the use of LLLT because the thyroid parenchyma is more preserved at this stage and better results can be achieved. In addition, safety demonstration is critical for professionals using LLLT in regions close to the thyroid for the treatment of other problems, such as muscle pain, inflammatory processes, and intraoral treatments.

## 5. Conclusion

The results suggest that LLLT is safe for the treatment of patients with CAT-induced hypothyroidism under the conditions specified herein, and therefore, subsequent applications may be considered for the purpose of maintaining or improving the obtained results. Future research will be important to corroborate these results.

## Figures and Tables

**Table 1:** Baseline characteristics of the patients included in this study (M2).

Features	L group (*n* = 25)	P group (*n* = 18)	*P* value
Mean age ± SD (years)	50.60 ± 10.07	49.10 ± 11.56	0.804
Female/male	25/1	18/0	
Mean disease duration ± SD (years)	11.33 ± 3.35	11.58 ± 3.48	0.601
Body mass (weight) ± SD (kg)	76.53 ± 16.24	74.74 ± 16.06	0.886
Body mass index (BMI)	30.87 ± 6.22	29.34 ± 4.96	0.570

**Table 2:** Comparison between the frequency of nodules in groups L and P 6 years after the conclusion of the RCT (M2).

Main outcome	Group L *n* = 25	Group P *n* = 18	Total *n* (%)	*P* value
Frequency of nodules	3 (12%)	3 (16.7%)	43 (100%)	0.663

Binomial test.

**Table 3:** Summary of the statistical analysis for the study groups (M2).

*Secondary outcomes*	Preintervention (M0)		*P* value	Postintervention (M2)		*P* value
	Group L (*n* = 25) Mean ± standard deviation	Group P (*n* = 20) Mean ± standard deviation		Group L (*n* = 23) Mean ± standard deviation	Group P (*n* = 20) Mean ± standard deviation	
LT4 dose (*μ*g/day)	93.48 ± 44.09	90.00 ± 40.88	0.791	94.50 ± 44.45	141.72 ± 52.62	*0.003* ^∗^
LT4 dose (*μ*g/kg/day)	1.32 ± 0.60	1.25 ± 0.55	0.693	1.31 ± 0.72	1.86 ± 0.53	*0.010* ^∗^
Total T3 (ng/mL)	124.04 ± 21.21	120.35 ± 20.02	0.563	107.25 ± 17.77	104.17 ± 18.66	0.589
Total T4 (*μ*g/dL)	9.92 ± 1.60	9.73 ± 1.95	0.738	10.27 ± 1.72	10.15 ± 2.33	0.848
Free T4 (ng/dL)	1.03 ± 0.15	1.03 ± 0.20	0.997	1.28 ± 0.21	1.28 ± 0.33	0.955
TSH (*μ*IU/mL)	2.65 ± 1.36	2.88 ± 1.41	0.579	1.82 ± 1.13	2.25 ± 1.91	0.397
Thyroid volume (cm^3^)	14.24 ± 9.78	16.32 ± 23.65	0.702	11.03 ± 5.97	14.80 ± 19.83	0.375
Mean of left and right ITA-PSV (cm/s)	28.34 ± 10.95	27.31 ± 8.74	0.738	20.23 ± 6.68	17.60 ± 7.07	0.220
Mean of left and right ITA-RI mean	0.56 ± 0.07	0.57 ± 0.07	0.423	0.60 ± 0.08	0.64 ± 0.08	0.351

*t*-test for independent samples; ^∗^*P* < 0.05.

**Table 4:** Comparison between the proportions of patients with normal and abnormal thyroid volume between groups L and P at M2.

Volume	Group L *n* (%)	Group P *n* (%)	Total *n* (%)	*P* value
Normal volume	18 (72)	7 (61)	25 (58.1)	0.030^∗^
Abnormal volume (increased or reduced)	7 (28)	11 (39)	18 (41.9)	
Total	25 (58.10)	18 (41.9)	43 (100)	

Chi-squared test.

**Table 5:** Comparison of the proportions of patients with normal and abnormal TVP between the L and the P groups at M2.

Vascularization	Group L *n* (%)	Group P *n* (%)	Total *n* (%)	*P* value
Normal vascularization	6 (12)	2 (6)	8 (58.1)	0.408
Abnormal vascularization (patterns I, III, and IV)	44 (88)	34 (94)	78 (41.9)	
Total	50 (58.1)	36 (41.9)	86 (100)	

Chi-squared test.
